# Prevalence and Practice Domains of Advanced Practice Nurses Among Participants in the Latin American Nursing Leadership School: A Cross-Sectional Study

**DOI:** 10.3390/healthcare13192515

**Published:** 2025-10-03

**Authors:** Patricia Rebollo-Gómez, Esperanza Barroso-Corroto, Joseba Rabanales-Sotos, Ángel López-González, José Alberto Laredo-Aguilera, Juan Manuel Carmona-Torres

**Affiliations:** 1 Nursing Development Foundation FUDEN, 28013 Madrid, Spain; patriciarebollo@fuden.es; 2 Faculty of Physiotherapy and Nursing, University of Castilla-La Mancha, 45005 Toledo, Spain; esperanza.barroso@alu.uclm.es (E.B.-C.); josealberto.laredo@uclm.es (J.A.L.-A.); juanmanuel.carmona@uclm.es (J.M.C.-T.); 3 Multidisciplinary Research Group in Care (IMCU), University of Castilla-La Mancha, 45005 Toledo, Spain; 4 Castilla-La Mancha Institute of Health Research (IDISCAM), 45004 Toledo, Spain; 5 Faculty of Nursing of Albacete, University of Castilla-La Mancha, 02071 Albacete, Spain; angel.lopez@uclm.es

**Keywords:** advanced practice nursing, Latin America, health services administration, education, licensure, nursing, health policy

## Abstract

**Aims:** This study aimed to determine the prevalence of nurses in a Latin American leadership school who meet advanced nurse standards. **Design:** A descriptive cross-sectional study was conducted. **Methods:** Data were collected between January and November 2024 from a total of 92 participants from the Latin American Leadership School of FUDEN-FEPPEN (Foundation for the Development of Nursing—Pan American Federation of Professional Nurses). The response rate was 13%. The Spanish version of the APRD (advance practice role delineation) was validated in Spanish. The study was approved by the Social Ethics Committee of UCLM (Universidad Castilla-La Mancha). Inference analysis was performed to examine factors associated with advanced practice domains. **Results:** A total of 92 nurses participated in the study. Among the participants, 35.86% (33 nurses) met the requirements for advanced practice nurses and the minimum training required by the International Council of Nurses. Nurses in both primary care and specialized care perform more advanced practice activities in direct care; however, nurses practicing teaching and research perform more advanced practice activities in the indirect practice domains (training, research and teaching). **Conclusions:** The percentage of nurses participating in the Latin American leadership school who met the standards was determined, with the most frequent domains those related to direct care, such as expert care planning, integrated care, and inter-professional collaboration. **Implications for the profession and patient care:** To our knowledge, this is the first study that describes the profile of advanced practice nurses in the Latin American context. This study shows that advanced practice activities exist and are practiced, but there is no clear delimitation or regulation of these activities. **Reporting method:** The study was conducted following the STROBE guidelines. **Public contribution:** This study did not include patient or public involvement in its design, conduct, or reporting.

## 1. Introduction

The development of advanced practice nurses (APNs) in Latin America and the Caribbean has been driven in response to improved access to health services, particularly in rural and underserved populations [1]. The origin of APNs in the United States (USA) can be traced back to the early 20th century and was influenced by a combination of sociopolitical and professional forces, such as territorial expansion and the needs of rural populations [2]. The historical context of the APN in Latin America and the Caribbean follows this same trajectory. However, factors such as resource disparities, variations in health structure, and different local policies influence the pace and extent of APN implementation in each region [3,4].

The implementation of these advanced roles has occurred unevenly in time, concept and content, with very disparate barriers and driving forces: from the shortage of doctors, the transformation of the needs of the population, the current crisis of the health system, or the improvement in the professional practice of nurses [2,5]. Countries such as Brazil have taken significant steps in the recognition of specialized nursing roles [6]. However, this recognition varies greatly by region, with some countries lacking formalized roles and regulatory frameworks, leading to inconsistencies in practice and service delivery [4,7,8].

The shortage of health professionals in Latin America and the Caribbean has been an impetus for the advancement of APNs in this region. In fact, the WHO (World Health Organization) forecasts a global shortage of 12.9 million doctors, nurses and midwives by 2035 [9].

This situation is coupled with the increasing prevalence of chronic diseases, integrating APN as a possible solution to improve healthcare delivery and coverage [10,11,12]. Given the shortage of health professionals, the clear implementation of the roles and recognition of nurses continues to shape the future of nursing in Latin America and the Caribbean, which needs a more structured approach to APN [13].

### Background

Since 2017, strategies to standardize advanced practice roles have been promoted in several Latin American and Caribbean countries, inspired by successful models such as those in the United States (US) and Europe [11]. This standardization seeks not only to clarify the scope but also to address feasibility in training and regulatory frameworks. Recently, Minosso et al. [14] conducted a methodological study of the Advance Practice Role Delineation (APRD) instrument [15], in which they demonstrated its stability and reliability through the evaluation of psychometric properties such as internal consistency, test–retest reliability, and construct validity. This instrument was also validated in Spanish by Sevilla Guerra et al. [16] with a sample of primary care nurses, confirming its linguistic and cultural applicability.

The APN implementation strategy in Latin America is focused on primary care [4]. Countries such as Brazil and Chile have master’s degree programs in nursing with great potential for the implementation of APNs [17]. In Brazil, a critical point in the recognition of APN was the introduction of the role of geriatric nurse specialists, who were granted diagnostic authority and autonomy in treatment [1,4], although it should be noted that APN roles are at an early stage of development in Latin America and the Caribbean [18,19].

The regulatory framework for APNs in Latin America is characterized by a complex interplay between each country’s national legislation, professional councils and associations, and the capacity of the local health system [20]. The Regional Forum for the Advancement of Nursing in the Americas [21] highlighted the need to form a common working group to establish a definition of APN adapted to the entire region on the basis of the criteria established by the ICN [22].

Similarly, the Pan American Health Organization (PAHO) presents key action points and calls for consideration of the importance of investing in nursing education, employment, leadership and practice [23].

Legislation in some Latin American countries presents significant barriers to the development and recognition of nurse practitioner roles. The scarcity of laws, in turn, limits the availability of academic institutions capable of offering training programs in advanced practice [20]. In Brazil, for example, they are regulated by a complex system involving different actors, such as federal, state and professional councils, creating inconsistencies in practice and limiting the scope of action of the APN [9]. In contrast, other countries, such as Ecuador, lack accredited APNs outside those recognized by US agencies, which affects their applicability and scope of advanced practice standards within the health system [4,8,24,25].

The training curricula for direct care nurses and specialists in Latin America vary from country to country. This impacts the quality of training and role recognition and thus affects the effectiveness of nurse practitioners within the healthcare system [4,8,12,26]

In a global study published by Wheeler et al. [9], there was evidence of formal education in the countries included in the study, but the depth of these programs differed markedly. Wheller reported that countries such as Australia, Canada, The Netherlands, the United Kingdom (UK) and the USA have more than ten APN education programs, whereas others have fewer options and requirements, offering alternative pathways such as the baccalaureate or advanced diploma.

As described above, we encounter important barriers that make it difficult to study the prevalence and distribution of APNs in Latin America. Moreover, to our knowledge, there are practically no known updated studies (conducted in the last 5 years) that analyze their prevalence. Minosso et al. [14] conducted a clinical validation of an instrument in 207 nurses active in primary care.

Therefore, this study aimed to analyze the prevalence of nurses enrolled in the Latin American leadership school who meet advanced practice nurse (APN) standards and to examine the associations between sociodemographic and professional characteristics and the scope and domains of advanced practice.

## 2. Methods

### 2.1. Design

A descriptive cross-sectional study with exploratory inferential analyses was conducted to explore the extent to which advanced practice nursing standards were met by nurses in the context of this study. The data collection period was between January 2024 and November 2024. This descriptive study was conducted following the standards of the Strengthening the Reporting of Observational Studies in Epidemiology (STROBE) guidelines.

### 2.2. Participants

The Foundation for the Development of Nursing (FUDEN) is an organization founded in 1989 that fosters the talent and competencies of nurses. It carries out training, research, and scientific dissemination activities, promoting innovation in care processes that facilitate and improve people’s lives. The Pan American Federation of Nursing Professionals (FEPPEN) is a nongovernmental organization under private law, with no nonprofit, religious, political, or partisan aims, constituted by national organizations of nursing professionals from Latin American and Caribbean countries. Both organizations, FUDEN and FEPPEN, signed an agreement in 2021 through which both parties committed to supporting and promoting activities of common interest within the field of nursing and health development aimed at fulfilling the objectives of both institutions. This collaboration has been realized in a leadership training program framed within the FUDEN-FEPPEN Latin American School of Leadership. The participants were the users of a leadership training plan included in the FUDEN Latin America School of Nursing, with free and open access available at https://www.fuden.es/escuela-liderazgo/ (accessed on 30 November 2024).

A convenience sampling method was used, including all nurses enrolled in the FUDEN-FEPPEN leadership program. The inclusion criteria were to be a nurse and to be carrying out one of the training activities during the data collection period. The participants were sent an email informing them of their participation. The GRANMO Calculator (v 7.12 April 2012) was used to calculate the sample size for population estimation. Taking as a reference a population of 705 nurses in the Latin American leadership school FUDEN-FEPPEN, a calculated sample size of 86 individuals is sufficient to estimate, with a confidence level of 95% and a precision of +/−10 percentage units, a population percentage expected to be approximately 50% (situation of maximum uncertainty due to lack of previous studies). A loss to follow-up rate of 10% was estimated. Follow-up reminders were sent via email to improve the response rate.

### 2.3. Instruments

Data were collected between January and November 2024 via the Spanish version of the Modified Advance Practice Nurse Role Delineation (APRD) [27] validated by Sevilla et al. [16], which has previously demonstrated acceptable reliability and validity for their use [28].

The instrument consists of two parts: one for collecting sociodemographic data and the other for instrument analysis. The sociodemographic variables collected in the study included age, sex, years of professional experience, educational level, area of working practice, usual working sector and region of work.

The APRD includes 38 items grouped into six domains of advanced practice. These domains are categorized into direct care (Domain 1: Expert care planning; Domain 2: Integrated care; Domain 3: Interprofessional collaboration) and indirect care (Domain 4: Education; Domain 5: Research and Evidence-Based Practice; Domain 6: Professional Leadership). The participants were asked to complete a five-point Likert scale (4 = Very much; 3 = Quite a lot; 2 = Somewhat; 1 = Hardly at all; 0 = Not at all) on how much time they spent on each of the indicated activities. The questionnaire provides a score ranging from 0 to 152 points, which is the sum of all the items, and a score for each dimension, which is the measure of the items corresponding to that domain. No weighting was applied in the scoring process.

The overall Cronbach’s α was 0.957. The Cronbach’s α values for each domain were as follows: Domain 1 (Expert Care Planning): 0.927; Domain 2 (Integrated Care): 0.940; Domain 3 (Interprofessional Collaboration): 0.879; Domain 4 (Education): 0.861; Domain 5 (Research and Evidence-Based Practice): 0.913; and Domain 6 (Professional Leadership): 0.905.

The cutoff scores presented in Table 1 were derived from the original validation study by Sevilla Guerra et al. [16], in which the authors calculated the average scores obtained by nurses identified as meeting advanced practice criteria. These empirical thresholds were established to differentiate between general and advanced practice levels across each domain on the basis of the distribution of responses in the Spanish sample.

### 2.4. Procedure

The researchers created a form located on the Fuden digital platform Moodle (https://campusinclusiva.fuden.es/ accessed on 30 November 2024). The questionnaire was administered at the FUDEN-FEPPEN leadership school, and the participants were contacted and asked to participate in the study. When entering the questionnaire, the participants had to read the information sheet and sign the informed consent form before accessing the questionnaire. The user had easy and direct access to the platform. The scale was placed at the top of the page to make it easier to see and to make it easier to fill in. In addition, the user was provided with information about the present study in several of the school’s training pills.

### 2.5. Ethical Considations

The study was approved by the Social Research Ethics Committee of the University of Castilla-La Mancha with reference number CEIS-722990-Z2G3. The participants received the study information sheet and signed the informed consent form before starting the study. The anonymity of the participants was guaranteed in the reporting of results and in the analysis of the research.

### 2.6. Statistical Analysis

The results of the statistical analysis are presented as the means and standard deviations for continuous variables and as counts (%) for categorical variables. An inferential analysis was performed to compare the performance of nurses according to their current position and level of training: (a) in the case of qualitative variables, a comparison of the proportions of categorical variables was performed via chi-square tests for contingency tables; (b) for quantitative variables, the goodness of fit to a normal distribution was determined first (using the Shapiro–Wilk test), and the homogeneity of variances was tested (using Levene’s test). If the data fitted a normal distribution, parametric tests (Student’s *t* test) were used. Otherwise, corresponding nonparametric tests (Mann–Whitney U) were applied. A logistic regression analysis was performed to evaluate the relationship between the dichotomous dependent variable and the independent variables, allowing us to estimate the probability of the event of interest occurring and control for possible confounding factors. All significance tests were considered at *p* < 0.05. All analyses were performed with SPSS version 28 (IBM Corp. Armonk, NY, USA), licensed by the University of Castilla-La Mancha.

## 3. Results

Finally, 92 nurses from the Latin American leadership school FUDEN-FEPPEN participated in the study, for a response rate of 13%. The sociodemographic data and overall characteristics of the sample are shown in Table 2. The age range was 27–63 years, with a mean age of 42.51 years (SD: 9.267). Among the participants, 90.2% were women, had been working in their current job for an average of 10.87 years (SD 7.85), had a master’s degree (47.8%), worked in hospital care (54.4%) and worked in the public sector (83.7%).

The normality of the total APRD instrument score and each domain was assessed via the Shapiro–Wilk test. The results revealed that both the overall score (*p* < 0.001) and the six domains had distributions that differed significantly from the normal distribution (Dom 1 *p* = 0.006; Dom 2 *p* = 0.001; Dom 3 *p* = 0.008; Dom 4 *p* < 0.001; Dom 5 *p* = 0.004; Dom 6 *p* = 0.002). The variables age (W = 0.956, *p* = 0.039) and years in the current position (W = 0.909, *p* < 0.001) do not meet the assumption of normality. Therefore, it is assumed that the data do not follow a normal distribution, and nonparametric tests were applied.

A total of 35.86% of the sample (33 nurses) met the requirements to be APN, fulfilling both the criteria of the definition of APN in the instrument used and the minimum training required by the ICN by certifying a master’s or doctoral degree. The mean APRD score was 5.726 (95% CI: 5.565–5.888), with a standard deviation of 0.692. The average scores for each domain can be found in Table 3.

Table 4 shows the scope of the APN standards according to the sociodemographic variables. There were 2 nurses who, not having a master’s or doctoral degree, could not be included as APNs despite meeting the advanced practice standards. A statistically significant association was found only for the level of studies.

Table 5 shows the performance of APN activities according to the area of professional practice and level of study.

A statistically significant association was found between the area of professional practice and one of the APN practice domains: comprehensive care (*p* < 0.001).

Primary care nurses perform more advanced practice activities in the direct care domain, with values above 90% in the domains of expert care planning and comprehensive care. This trend remains the same for nurses in specialized hospital care (including nurses who carry out their practice in special hospital services such as the ICU, operating theater and/or emergency room), who obtain 96.9% in expert care planning, 96.9% in integrated care and 93.8% in interprofessional collaboration. However, nurses in hospitals score 100% in terms of expert care planning, comprehensive care, interprofessional collaboration and indirect care, such as research and evidence-based practice.

In the indirect care domain, 80.0% and 84.4% of the participants were in primary care and specialized hospital care, respectively. This trend differs for nurses who practice in management, with 100.0% of nurses in the training domain and 100.0% in the research domain, but the percentage decreases to 71.4% in professional leadership. Research nurses scored 100% in terms of research, education and professional leadership. Nurses working in teaching and research roles demonstrated greater engagement in indirect practice domains, including training, research, and professional leadership, than did nurses in clinical care roles.

Table 6 shows the performance of APN activities as a function of educational level. There was no significant relationship with any of the APN domains, confirming that there is no specific pattern of APN activity in these domains according to the level of education. However, it is worth noting that to achieve standards for advanced practice, a master’s or doctoral degree is needed. Specifically, 100% of nurses with a doctoral degree achieve comprehensive care, interprofessional collaboration, education and professional leadership.

The chi-square test analysis revealed a significant relationship between Domain 4 (education and training) scores and two relevant sociodemographic variables. First, educational level is statistically significantly associated with Domain 4 scores, indicating that professionals with greater academic training tend to be more involved in activities related to education and training. Similarly, the area of work showed a significant association with this domain, indicating that the type of usual practice (care, teaching, or management) conditions participation in educational and training competencies.

Finally, of the 92 participating nurses, 33 (35.86%) met the requirements to be APNs, not only because they met the criteria for the definition of APNs in the instrument used but also because they met the minimum training required by the ICN by certifying a master’s or doctoral degree. In the case of a master’s degree, 29 of them, i.e., 82.9%, met the requirements, and in the case of a doctorate, 4 of them, i.e., 5.7%, could be considered APNs.

The Mann–Whitney U test was used to examine differences in APRD scores between men and women. No statistically significant differences were observed (U = 450.5, Z = 1.192, *p* = 0.233). Similarly, the test was applied to compare the distributions of age between participants who met the APN standards and those who did not. Again, no significant differences were found (U = 751, N = 79, Z = 0.265, *p* = 0.791), indicating that the distributions of both groups were comparable. Finally, the Mann–Whitney test was conducted to compare the number of years in the current position between participants who met the APN criteria and those who did not. No significant differences were detected (U = 412, N = 61, Z = −0.684, *p* = 0.494), suggesting that the distributions of the two groups were similar.

According to the logistic regression analysis, only age was significantly associated with meeting the standards for being an EPA (OR = 0.86; 95% CI: 0.75–0.99). The other variables did not reach statistical significance, probably because of the small number of cases in certain categories, which limited the power of the analysis.

## 4. Discussion

In this study, we identified a 35.86% (*n* = 33) prevalence of APNs in the Latin American leadership school, who were aware of their scope and patterns of advanced nursing practice in a nurse leadership training program, both for their role definition and for reaching the minimum required training. Importantly, the findings of this study reflect the experiences of nurses enrolled in the FUDEN–FEPPEN leadership program rather than the broader nursing workforce in Latin America and the Caribbean. As such, the results should be interpreted within the context of a sample with a particular interest in leadership and postgraduate development.

A previous study [28] revealed that 269 nurses, i.e., 22.2% of nurses, met the requirements to be APNs. The difference with respect to our study may be explained by the fact that the nurses included in the sample had a higher level of education; specifically, 47.8% had a master’s degree, and 6.5% had a doctorate, which is a requirement of the ICN to be considered an advanced practice nurse. In addition, our sample is limited to nurses enrolled in a leadership school in Latin America and in a postgraduate training plan that specifically focuses on the competencies of advanced practice nurses. This leads us to assume that the sample has a special interest in leadership development and postgraduate educational development. All the registered nurses are members of one of the nursing associations affiliated with the Pan American Federation of Professional Nurses (FEPPEN), so it may be that they are highly interested in professional development.

In terms of practice domains, the nurses in the present study achieved higher standards of advanced practice in the direct care domain: expert care planning, integrated care and interprofessional collaboration, with values even above 90%, decreased slightly in the training, research and professional leadership domains. Similarly to our findings, APNs scored higher on average in the direct care domains in the context of Spain [28], New Zealand [29] and Australia [30], with the lowest scoring domains being research, training and professional leadership. These findings are aligned with those of previous international studies [6,29,31]. In the Finnish context, advanced practice nurses [32] have lower scores for the direct clinical care domain than generalist nurses do.

In the Saudi Arabian context [33], the findings showed that nurses, regardless of their current role, scored high in all practice domains, with the highest average score in clinical care and the lowest score in leadership. These findings agree with the results presented in the present study, with the lowest score (75%) in the clinical leadership domain and the highest score (93.5%) in the comprehensive care domain. This is evidence that both in Saudi Arabia and in Latin America and the Caribbean, advanced practice functions and activities are practiced; nevertheless, there is no clear delineation of these functions and activities according to a unified scope of practice [25].

Notably, higher domain scores in indirect practice (training and leadership) were associated with higher education, in the present study, for nurses with master’s or doctoral degrees, as well as for teaching, management and research positions. These findings are similar to those of a study published in New Zealand [29,34], where a very specific role in that context (Clinical Nurse Especialist (CNS role), equivalent to a clinical nurse consultant role and with higher consultant education) was associated with higher scores in these domains.

Among the sample, the most represented group was nurses practicing specialized hospital care, 60% of whom met the criteria to be considered APNs. This is evidence of the demand for nurses specializing in chronic, acute, and especially complex patients [35]. However, in Latin America, it has been driven in response to improved healthcare access [1] and the scarcity and disparity of resources [3].

The results shown can help lay the knowledge base for advanced practice in Latin America and the Caribbean and provide guidelines for the formal design of its role in future implementation. In Latin America and the Caribbean, there is currently no regulation or accreditation for APNs, which are in an early stage of development in Latin America [25].

In the international context, since 2008, the USA has taken the first steps in its regulation through a model that includes different licenses [36]. In the case of Canada, the government has given nursing professionals the responsibility to self-regulate through the Canadian Nurses Association [37]. In the Latin American context, countries such as Brazil and Chile already have master’s programs that could serve as academic accreditations for possible APN accreditations [17].

ICN has presented case studies on APNs around the world that show that these roles lead to new, innovative and even entrepreneurial healthcare delivery in a variety of settings, addressing integrated health needs [38]. This figure has a direct impact on health outcomes and user satisfaction and, therefore, on the quality of care provided, but we must continue to make progress in this figure by unifying its concept and implementation [39].

Importantly, in Latin America, research on advanced practice nursing remains limited; however, countries such as Brazil [40,41] and Chile [42] have implemented master’s programs that serve as potential academic pathways for advanced practice roles, although these programs remain largely unregulated at the national level. This contrasts with international contexts where regulatory and educational frameworks are more established. For example, in the United States and Canada [37], advanced practice roles are supported by formal licensure or self-regulation through professional associations, whereas in Australia and New Zealand [29], advanced practice is embedded in postgraduate education and national health policy. These differences highlight how the presence—or absence—of regulatory structures and standardized educational models directly shapes the scope of practice, recognition, and professional development opportunities for advanced practice nurses across different regions.

Given this situation, we must bear in mind that the role of the APN is at an early stage of development in Latin America and the Caribbean [18,19,25]. Therefore, further strengthening of the education and health system is needed to support and regulate the workforce.

### 4.1. Study Limitations

Among the limitations of this study, the questionnaire used for data collection was self-administered. Given that the study population includes nurses undergoing leadership school training, it may be that they are more trained and motivated than other nurses are to meet the APN standards, which introduces potential selection bias. The relatively low response rate (13%) raises the possibility of nonresponse bias. The participants who chose to complete the questionnaire may differ systematically from those who did not, for example, in terms of motivation, engagement with professional development, or leadership interest. Because only limited sociodemographic information was available for nonrespondents, it was not possible to conduct a formal nonresponse analysis.

The cross-sectional design does not allow causal inferences, and the reduced size of some subgroups limits the possibility of more detailed analyses. Furthermore, inconsistencies previously detected in APRD scoring could have led to potential misclassification. The lack of previous APN studies in Latin America and the small sample size made it impossible to analyze the situation for each country.

A sufficient sample size was recruited to guarantee the validity and reliability of the results, and this is the first study to be carried out in Latin America via a validated questionnaire with a population from the entire national territory.

### 4.2. Research Implications

Future research should address the limitations identified in this study, including the need for larger and more representative samples across different Latin American countries, as well as the use of longitudinal designs to better understand causal relationships.

The findings of this study provide preliminary insights into the profile and distribution of APN activities among participants in a Latin American leadership program. These results highlight several areas for future research. First, studies with larger and more representative samples across countries and healthcare settings are needed to better understand the prevalence and scope of APN in the region. Second, longitudinal research could explore the development of competencies over time, including how educational attainment and professional roles influence engagement in direct and indirect practice domains. Third, further validation of the modified APRD instrument in diverse Latin American contexts is recommended to ensure its cultural and psychometric appropriateness. Finally, the domain mapping approach used in this study offers a framework for evaluating APN activities systematically, which can inform the design of educational programs, professional development initiatives, and future efforts to define advanced practice roles regionally.

## 5. Conclusions

The prevalence of nurses who met the standards for the APN was 35.86% among participants of the FUDEN–FEPPEN Latin America leadership school. The nurses also met the minimum training required by the ICN by certifying possession of a master’s or doctoral degree, with nearly half holding a master’s degree and a smaller proportion holding a doctoral degree. Consistent with international trends, nurses perform more advanced practice activities in direct care in both primary care and hospital care, whereas less advanced practice activities are performed in indirect care domains (training, research and teaching). This trend differs for nurses practicing in the teaching and research domains by meeting standards in the indirect practice domains (training, research and teaching). Notably, higher standards in indirect practice were more often achieved by nurses working in teaching, management, or research roles.

Importantly, these findings describe the profile of nurses engaged in a leadership program and cannot be generalized to the broader nursing workforce in Latin America and the Caribbean. Nevertheless, the results provide valuable exploratory insights into advanced practice nursing in the region, with patterns comparable to those reported internationally in contexts with more consolidated educational and regulatory frameworks. As in many other countries, the competencies of nurses are continuously evolving due to the increasing demand and general shortage of health professionals.

Our results show some similarities with those of other countries that are in more developed stages of APN implementation. Therefore, the present study provides evidence that advanced practice activities exist and are practiced without a clear delimitation and regulation of these activities. Despite the existence of such activities, the ambiguity of functions and variations in education could hinder the development and implementation of the role of APNs. The domain mapping of APN activities described in this study has practical value for designing educational and regulatory pathways. However, it is essential to recognize the exploratory nature of this study. Further research with representative sampling (e.g., by country or sector) is needed to confirm these findings. Advanced practice nurses are growing worldwide and are described as ‘sleeping giants’ because of their potential to increase access to healthcare and the quality of the healthcare system. Therefore, governments must reorient their policies to support their growing workforce, particularly APNs, as an effective resource in promotion, prevention, care management and partnerships.

## Figures and Tables

**Table 1:** Average minimum score for each domain to achieve the EPA.

Domain 1: Expert Care Planning	Domain 2: Integrated Care	Domain 3: Interprofessional Collaboration	Domain 4: Education	Domain 5: Research and EBP (Evidence-Based Practice)	Domain 6: Professional Leadership
2	2	2	2	1.7	1.7

**Table 2:** Sociodemographic characteristics of the participants (*n* = 92).

Variables	*n* (%)
Sex - Female - Male	83 (90.2) 9 (9.8)
Level of education - Diploma - Bachelor’s degree - Expert - Master’s degree - Doctorate - Other	2 (2.2) 27 (29.4) 11 (12) 44 (47.8) 6 (6.5) 2 (2.2)
Area of usual practice - Primary Care - Hospital - Social and healthcare - Management - Teaching - Research - Special service - Other	15 (16.3) 50 (54.4) 2 (2.2) 7 (7.6) 9 (9.8) 2 (2.2) 1 (1.1) 6 (6.5)
Usual sector of work - Public sector - Private - NGO - Other	77 (83.7) 10 (10.9) 3 (3.3) 2 (2.2)
Region of work - National - Regional - Metropolitan - Rural	23 (25) 45 (48.9) 14 (15.2) 10 (10.9)
	Mean (SD)
Age	42.51 (9.267)
Number of years in current job	10.87 (7.854)

Scope of advanced practice standards.

**Table 3:** Median scores and SDs for each APRD domain.

Domain 1	Domain 2	Domain 3	Domain 4	Domain 5	Domain 6
3.029 (±0.834)	3.144 (±0.752)	3.004 (±0.755)	3.151 (±0.780)	2.756 (±0.881)	2.934 (±0.798)

**Table 4:** Relationships between advanced practice standards and sociodemographic variables.

	APN		χ^2^ (*p* Value)
	Non (%)	Yes n (%)	
Sex - Female - Male	51 (89.5) 6 (10.5)	32 (91.4) 3 (8.6)	0.094 (0.759)
Level of education - Diploma - Bachelor’s degree - Expert - Master’s degree - Doctorate - Other	2 (3.5) 27 (47.4) 11 (19.3) 15 (26.3) 2 (3.5)0	00029 (82.9) 4 (11.4) 2 (5.7)	44.399 (<0.001 **)
Area of usual practice - Primary Care - Hospital - Social and healthcare - Management - Teaching - Research - Special service - Other	9 (15.8) 29 (50.9) 2 (3.5) 6 (10.5) 6 (10.5)01 (1.8) 4 (7)	6 (17.1) 21 (60)01 (2.9) 3 (8.6) 2 (5.7)02 (5.7)	8.169 (0.417)
Usual sector of work - Public sector - Private - NGO - Other	45 (78.9) 8 (14) 3 (5.3) 1 (1.8)	32 (91.4) 2 (5.7) 1 (0.4)0	3.748 (0.290)
Region of work - National - Regional - Metropolitan - Rural	14 (24.6) 27 (47.4) 9 (15.8) 7 (12.3)	9 (25.7) 18 (25.7) 5 (14.3) 3 (8.6)	0.391 (0.942)
	Mean (SD)	Mean (SD)	
Age	42.38 (9.773)	42.72 (8.485)	
Number of years in current job	11.59 (8.418)	9.96 (7.133)	

** *p* < 0.001.

**Table 5:** Performance of advanced practice activities according to the area of practice.

	Expert Care Planning			Comprehensive Care			Interprofessional Collaboration			Training			Research and Evidence-based Practice			Professional Leadership		
Area of Usual Practice	Yes	No	Χ^2^ (p)	Yes	No	Χ^2^ (p)	Yes	No	Χ^2^ (p)	Yes	No	Χ^2^ (p)	Yes	No	Χ^2^ (p)	Yes	No	Χ^2^ (p)
	*n* (%)	*n* (%)		*n* (%)	*n* (%)		*n* (%)	*n* (%)		*n* (%)	*n* (%)		*n* (%)	*n* (%)		*n* (%)	*n* (%)	
Primary Care (*n* = 15)	14 (93.3)	1 (6.7)	13.009 (0.112)	15 (100)	0 (0.0)	25.208 (0.001 **)	13 (86.7)	2 (13.3)	4.562 (0.803)	10 (66.7)	5 (33.3)	18.157 (0.020 *)	13 (86.7)	2 (13.3)	8.736 (0.365)	12 (80.0)	3 (20.0)	14.711 (0.065)
Special care/Hospital (*n* = 32)	31 (96.9)	1 (3.1)		31 (96.9)	1 (3.1)		30 (93.8)	2 (6.3)		31 (96.9)	1 (3.1)		28 (87.5)	4 (12.5)		27 (84.4)	5 (15.6)	
Sociosanitary (*n* = 2)	1 (50.0)	1 (50.0)		1 (50.0)	1 (50.0)		2 (100)	0 (0.0)		2 (100)	0 (0.0)		2 (100)	0 (0.0)		0 (0.0)	2 (100)	
Teaching (*n* = 9)	8 (88.9)	1 (11.1)		8 (88.9)	1 (11.1)		8 (88.9)	1 (11.1)		8 (88.9)	1 (11.1)		6 (66.7)	3 (33.3)		6 (66.7)	3 (33.3)	
Management (*n* = 7)	5 (71.4)	2 (28.6)		4 (57.1)	3 (42.9)		7 (100)	0 (0.0)		7 (100)	0 (0.0)		7 (100)	0 (0.0)		5 (71.4)	2 (28.6)	
Hospitalization (*n* = 18)	18 (100)	0 (0.0)		18 (100)	0 (0.0)		18 (100)	0 (0.0)		18 (100)	0 (0.0)		18 (100)	0 (0.0)		10 (55.6)	8 (44.4)	
Research (*n* = 2)	2 (100)	0 (0.00)		2 (100)	0 (0.0)		2 (100)	0 (0.0)		2 (100)	0 (0.00)		2 (100)	0 (0.0)		2 (100)	0 (0.0)	
Other (*n* = 6)	5 (83.3)	1 (16.7)		6 (100)	0 (0.0)		5 (83.3)	1 (16.7)		6 (100)	0 (0.0)		5 (83.3)	1 (16.7)		6 (100)	0 (0.0)	
Special service (*n* = 1)	1 (100)	0 (0.0)		1 (100)	0 (0.0)		1 (100)	0 (0.0)		1 (100)	0 (0.0)		1 (100)	0 (0.0)		1 (100)	0 (0.0)	
TOTAL (*n* = 92)	85 (92.4)	7 (7.6)		86 (93.5)	6 (6.5)		86 (93.5)	6 (6.5)		85 (92.4)	7 (7.6)		82 (89.1)	10 (10.9)		69 (75.0)	23 (25.0)	

* *p* < 0.05, ** *p* < 0.001.

**Table 6:** Performance of APN activities according to level of education.

	Expert Care Planning			Comprehensive Care			Interprofessional Collaboration			Training			Research and Evidence-Based Practice			Professional Leadership		
Level of Education	Yes	No	X^2^ (p)	Yes	No	X^2^ (p)	Yes	No	X^2^ (p)	Yes	No	X^2^ (p)	Yes	No	X^2^ (p)	Yes	No	X^2^ (p)
	*n* (%)	*n* (%)		*n* (%)	*n* (%)		*n* (%)	*n* (%)		*n* (%)	*n* (%)		*n* (%)	*n* (%)		*n* (%)	*n* (%)	
Diploma (*n* = 2)	2 (100)	0 (0.0)	2.075 (0.839)	2 (100)	0 (0.0)	3.509 (0.622)	2 (100)	0 (0.0)	2.031 (0.845)	2 (100)	0 (0.0)	11.714 (0.039 *)	2 (100)	0 (0.0)	2.482 (0.779)	1 (50.0)	1 (50.0)	4.103 (0.535)
Bachelor’s degree (*n* = 27)	25 (92.6)	2 (7.4)		26 (96.3)	1 (3.7)		24 (88.9)	3 (11.1)		21 (77.8)	6 (22.2)		23 (85.2)	4 (14.8)		20 (74.1)	7 (25.9)	
Expert (*n* = 11)	11 (100)	0 (0.0)		11 (100)	0 (0.0)		10 (90.9)	1 (9.1)		11 (100)	0 (0.0)		11 (100)	0 (0.0)		7 (63.6)	4 (36.4)	
Master’s degree (*n* = 44)	40 (90.9)	4 (9.1)		39 (88.6)	5 (11.4)		42 (95.5)	2 (4.5)		43 (97.7)	1 (2.3)		39 (88.6)	5 (11.4)		33 (75.0)	11 (25.0)	
Doctorate (*n* = 6)	5 (83.3)	1 (16.7)		6 (100)	0 (0.0)		6 (100)	0 (0.0)		6 (100)	0 (0.0)		5 (83.3)	1 (16.7)		6 (100)	0 (0.0)	
Other (*n* = 2)	2 (100)	0 (0.0)		2 (10.0)	0 (0.0)		2 (100)	0 (0.0)		2 (100)	0 (0.0)		2 (100)	0 (0.0)		2 (100)	0 (0.0)	
TOTAL (*n* = 92)	85 (92.4)	7 (7.6)		86 (93.5)	6 (6.5)		86 (93.5)	6 (6.5%)		85 (82.4)	7 (7.6)		82 (89.1)	10 (10.9)		69 (75.0)	23 (25.0)	

* *p* < 0.05.

## Data Availability

The minimum data set supporting this study’s findings is available within the manuscript. More information can be requested from the corresponding author upon reasonable request. The authors affirm that the methods used in the data analyses are suitably applied to their data within their study design and context, and the statistical findings have been implemented and interpreted correctly.
